# Transcriptome alterations of vascular smooth muscle cells in aortic wall of myocardial infarction patients

**DOI:** 10.1016/j.dib.2018.01.108

**Published:** 2018-02-06

**Authors:** Thidathip Wongsurawat, Chin Cheng Woo, Antonis Giannakakis, Xiao Yun Lin, Esther Sok Hwee Cheow, Chuen Neng Lee, Mark Richards, Siu Kwan Sze, Intawat Nookaew, Vladimir A. Kuznetsov, Vitaly Sorokin

**Affiliations:** a Department of Genome and Gene Expression Data Analysis, Bioinformatics Institute, Agency for Science, Technology and Research (A*STAR), Singapore 138671, Singapore; b Department of Biomedical Informatics, College of Medicine, University of Arkansas for Medical Sciences, Little Rock, AR 72205, USA; c Department of Surgery, Yong Loo Lin School of Medicine, National University of Singapore, Singapore 119228, Singapore; d Department of Cardiac, Thoracic and Vascular Surgery, National University Heart Centre, Singapore, National University Health System, Singapore 119228, Singapore; e School of Biological Sciences, Nanyang Technological University, Singapore 639798, Singapore; f Cardiovascular Research Institute, National University Heart Centre, Singapore, 119228, Singapore; g Department of Medicine, Yong Loo Lin School of Medicine, National University of Singapore, Singapore 119228, Singapore; h School of Computer Science and Engineering, Nanyang Technological University, Singapore 639798, Singapore

## Abstract

This article contains further data and information from our published manuscript [1]. We aim to identify significant transcriptome alterations of vascular smooth muscle cells (VSMCs) in the aortic wall of myocardial infarction (MI) patients. Microarray gene analysis was applied to evaluate VSMCs of MI and non-MI patients. Prediction Analysis of Microarray (PAM) identified genes that significantly discriminated the two groups of samples. Incorporation of gene ontology (GO) identified a VSMCs-associated classifier that discriminated between the two groups of samples. Mass spectrometry-based iTRAQ analysis revealed proteins significantly differentiating these two groups of samples. Ingenuity Pathway Analysis (IPA) revealed top pathways associated with hypoxia signaling in cardiovascular system. Enrichment analysis of these proteins suggested an activated pathway, and an integrated transcriptome-proteome pathway analysis revealed that it is the most implicated pathway. The intersection of the top candidate molecules from the transcriptome and proteome highlighted overexpression.

**Specifications Table**

**Table:** 

Subject area	Biology
More specific subject area	Genomics, Proteomics, Bioinformatics, Cardiovascular
Type of data	Tables, figures
How data was acquired	Microarray (Gene Titan Instrument, Affymetrix), mass spectrometry (LC-MS/MS system comprised of a Dionex Ultimate 3000 RSLC nano-HPLC system, coupled to an online Q-Exactive hybrid quadrupole-Orbitrap mass spectrometer (Thermo Scientific, Hudson, NH, USA)), RT-qPCR (QuantStudio™ 12K Flex system (Life Technologies; Thermo Fisher Scientific Inc, USA))
Data format	Raw, analyzed
Experimental factors	Laser capture microdissection, total RNA extraction and protein extraction from aortic tissues from surgical patients
Experimental features	Data analysis with Principal Component Analysis (PCA), Prediction Analysis of Microarray (PAM), Gene Ontology (GO), Ingenuity Pathway Analysis (IPA)
Data source location	Singapore
Data accessibility	Data is with this article.

**Value of the data**

• Combination of multiple technologies and bioinformatics analysis performed in this study reveals the molecular changes induced by myocardial infarction on aortic smooth cells in humans.
• The alterations of the VSMCs transcriptome are congruent with alterations at the protein levels. Both levels show notably the up-regulation of the superoxide dismutase (SOD) with the activation of superoxide radical degradation pathway.
• Differentially expressed genes and pathways identified in these comparisons may be used in future experiments investigating response in myocardial infarction.

## 1 Data

### 1.1 Clinical analysis

The characteristics of the myocardial infarction (MI) and non-MI samples undergoing transcriptomics and proteomics studies are presented in Table 1A, Table 1B respectively. The baseline demographic and clinical characteristics of samples undergoing transcriptomics study were compared with those of the samples from the proteomics study (Table 2). In addition, the characteristics of the transcriptomic MI and non-MI samples with those of the independent cohorts comprising additional MI and non-MI patients undergoing RT-qPCR were compared (Table 3, Table 4).

**Table 1A:** Demographic characteristics of MI and non-MI groups undergoing transcriptomics analysis.

**Characteristics**		**Transcriptomics**	**Transcriptomics**	***p*****-value**
		**MI**	**Non-MI**	
		**(n=17)**	**(n=19)**	
Ethnic	Chinese	12	10	0.557
	Malay	2	6	
	Indian	2	2	
	Others	1	1	
Gender	Male	14	16	0.881
	Female	3	3	
Age (Mean ± SD)		59.53 ± 8.28	59.68 ± 8.85	0.957
Ejection Fraction	Good (>45%)	11	13	0.292
	Fair (30–45%)	4	6	
	Poor (<30%)	2	0	
Smoking	No	8	9	0.985
	Yes	9	10	
Renal Impairment	No	15	19	0.124
	Yes	2	0	
Diabetes Mellitus	No	9	7	0.332
	Yes	8	12	
Hypertension	No	1	3	0.345
	Yes	16	16	
Hyperlipidaemia	No	0	0	–
	Yes	17	19	
Antihyperlipidemic Medication	No	0	0	–
	Yes	17	19	
Troponin I (µg/L)		12.20 ± 20.86	0.01 ± 0.004	<0.05
(Mean ± SD)		(n=15)	(n=4)

**Table 1B:** Demographic characteristics of MI and non-MI proteomics groups.

**Characteristics**		**Proteomics**	**Proteomics**	***p*****-value**
		**MI**	**Non-MI**	
		**n=25**	**n=25**	
Ethnic	Chinese	11	13	0.745
	Malay	8	7	
	Indian	5	5	
	Others	1	0	
Gender	Male	20	18	0.508
	Female	5	7	
Age (Mean ± SD)		60.88±12.34	61.68±8.26	0.789
Ejection Fraction	Good (>45%)	14	16	0.344
	Fair (30–45%)	9	9	
	Poor (<30%)	2	0	
Smoking	No	12	12	1
	Yes	13	13	
Renal Impairment	No	25	25	NA
	Yes	0	0	
Diabetes Mellitus	No	10	9	0.771
	Yes	15	16	
Hypertension	No	3	1	0.297
	Yes	22	24	
Hyperlipidaemia	No	1	0	0.312
	Yes	24	25	
Antihyperlipidemic Medication	No	4	1	0.157
	Yes	21	24	
Troponin I (µg/L)		19.54 ± 19.24	0.015 ± 0.006	<0.05
(Mean ± SD)		(n=22)	(n=9)

**Table 2:** Comparative demographic characteristics of transcriptomics and proteomics groups.

**Characteristics**		**Transcriptomics**	**Proteomics**	***p*****-value**
		**n=36**	**n=50**	
Ethnic	Chinese	22	24	0.404
	Malay	8	15	
	Indian	4	10	
	Others	2	1	
Gender	Male	30	38	0.41
	Female	6	12	
Age (Mean ± SD)		59.61 ± 8.47	61.28 ± 10.40	0.431
Ejection Fraction	Good (>45%)	24	30	0.708
	Fair (30–45%)	10	18	
	Poor (<30%)	2	2	
Smoking	No	17	24	0.943
	Yes	19	26	
Renal Impairment	No	34	50	0.092
	Yes	2	0	
Diabetes Mellitus	No	16	19	0.548
	Yes	20	31	
Hypertension	No	4	4	0.624
	Yes	32	46	
Hyperlipidaemia	No	0	1	0.393
	Yes	36	49	
Antihyperlipidemic Medication	No	0	5	0.051
	Yes	36	45	
Troponin I (µg/L)		9.63 ±19.09	13.87 ± 18.45	0.445
(Mean ± SD)		(n=19)	(n=31)

**Table 3:** Demographic characteristics of MI study group and MI validation group.

**Characteristics**		**MI Microarray**	**MI Validation**	***p*****-value**
		**(n=17)**	**(n=20)**	
Ethnic	Chinese	12	14	0.662
	Malay	2	4	
	Indian	2	2	
	Others	1	0	
Gender	Male	14	17	0.828
	Female	3	3	
Age (Mean ± SD)		59.53 ± 8.28	61.40 ± 7.88	0.487
Ejection Fraction	Good (>45%)	11	10	0.661
	Fair (30–45%)	4	7	
	Poor (<30%)	2	3	
Smoking	No	8	8	0.666
	Yes	9	12	
Renal Impairment	No	16	20	0.272
	Yes	1	0	
Diabetes Mellitus	No	9	6	0.157
	Yes	8	14	
Hypertension	No	1	4	0.211
	Yes	16	16	
Hyperlipidaemia	No	0	1	0.35
	Yes	17	19	
Antihyperlipidemic Medication	No	0	1	0.35
	Yes	17	19	
Troponin I (µg/L)		12.20 ± 20.86	20.94 ± 27.80	0.319
(Mean ± SD)		(n=15)	(n=17)

**Table 4:** Demographic characteristics of non-MI study group and non-MI validation group.

**Characteristics**		**Non-MI Microarray**	**Non-MI Validation**	***p*****-value**
		**(n=19)**	**(n=20)**	
Ethnic	Chinese	10	12	0.763
	Malay	6	6	
	Indian	2	2	
	Others	1	0	
Gender	Male	16	17	0.946
	Female	3	3	
Age (Mean ± SD)		59.68 ± 8.85	59.95 ± 8.34	0.924
Ejection Fraction	Good (>45%)	13	13	0.083
	Fair (30–45%)	6	3	
	Poor (<30%)	0	4	
Smoking	No	9	11 9	0.634
	Yes	10		
Renal Impairment	No	18	15	0.088
	Yes	1	5	
Diabetes Mellitus	No	7	8	0.839
	Yes	12	12	
Hypertension	No	3	4	0.732
	Yes	16	16	
Hyperlipidaemia	No	0	0	–
	Yes	19	20	
Antihyperlipidemic Medication	No	0	0	–
	Yes	19	20	
Troponin I (µg/L)		0.01 ± 0.004	NA	NA
(Mean ± SD)		(n=4)		

### 1.2 Gene expression data analysis and class prediction by Prediction Analysis of Microarray (PAM)

**Fig. 1:**
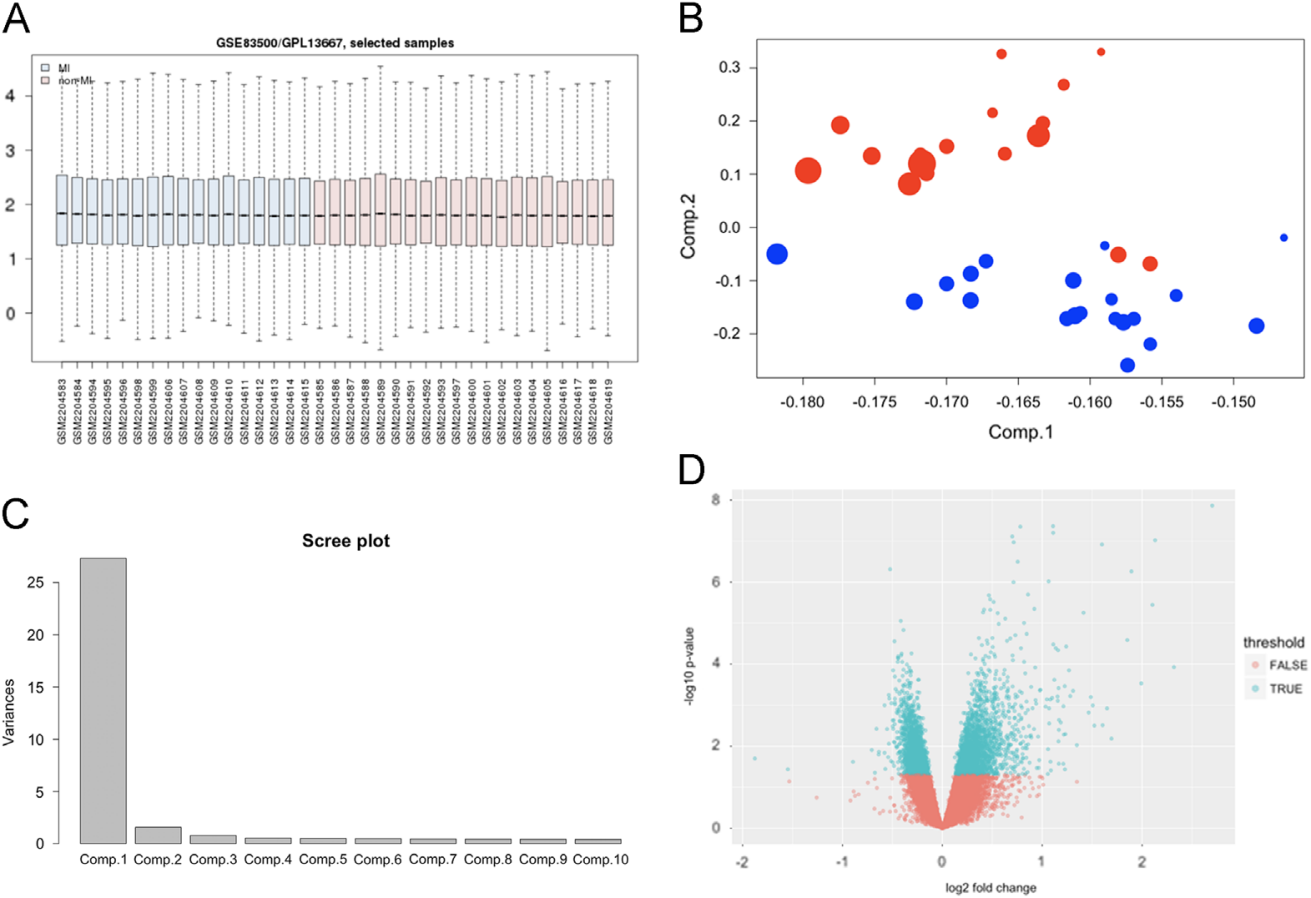
(A) Normalized data. (B) Pseudo three dimensional plot of PCA analysis of the 4,357 DEGs between MI (red) and non-MI (blue). The sizes of the dot represent the loading values of the Comp.3 that perpendicular on the Comp.1 and Comp.2 plane. (C) Scree plot shows the variances explained by the individual principle component. (D) Volcano plot of expression data. Green dot represents differentially expressed genes.

The samples were preprocessed through several steps, including quality assessment and outlier identification, normalization, batch effect correction and evaluation (Fig. 1). To interrogate differentially expressed genes between MI and non-MI we conducted gene-expression profiling using the Affymetrix U219 microarray platform. The R ‘limma’ package (https://www.bioconductor.org/help/workflows/arrays/) identified 4,357 probe sets, selected at a ‘limma’-defined *p*-value < 0.05. Based on this set of differentially expressed genes (DEGs), we performed principal component analysis (PCA) (Fig. 1).

To determine subgroup of genes distinguishing MI from non-MI subjects, we performed supervised PAM [2] and identified a set of differentially expressed genes (DEGs) that discriminated between the two subtypes at Wilcox FDR < 0.1 (Table 5).

**Table 5:** List of differentially expressed transcripts.

**Probe ID**	**Gene symbol**	**Up/down regulated in MI**	**wilcox**	**wilcox FDR**
11760204_x_at	CKMT1B	Upregulated inMI	0.00035204	0.00224576
11760991_a_at	CKMT1B	Upregulated in MI	0.00101536	0.00364739
11718483_s_at	UBE2N	Upregulated in MI	0.0001261	0.00150508
11752082_a_at	CDH12	Upregulated in MI	0.00010799	0.00137774
11744327_x_at	UBB	Upregulated in MI	1.4127E-06	0.00026136
11735320_a_at	RBMS3	Upregulated in MI	4.8014E-05	0.00093501
11743116_s_at	KPNB1	Upregulated in MI	4.2217E-06	0.0003841
11716989_s_at	PNRC2	Upregulated in MI	0.00053133	0.00252042
11761378_at	NAALADL2	Upregulated in MI	0.0006064	0.00276999
11754075_s_at	KRT222	Upregulated in MI	0.00053077	0.00252042
11718123_at	AIMP1	Upregulated in MI	0.00010799	0.00137774
11721001_at	HEXIM1	Upregulated in MI	0.00456914	0.00871434
11759202_s_at	C9orf41	Upregulated in MI	0.00019823	0.0016669
11758784_at	UBE3A	Upregulated in MI	0.00053077	0.00252042
11764120_at	–	Upregulated in MI	0.00017084	0.0016669
11729194_s_at	MYNN	Upregulated in MI	9.2259E-05	0.00137774
11757837_x_at	HNRNPA1	Upregulated in MI	0.00078736	0.0030992
11721747_a_at	ANKRD12	Upregulated in MI	0.00115015	0.00390417
11753179_s_at	FAM134B	Upregulated in MI	0.00046374	0.00245118
11757794_s_at	PAPD5	Upregulated in MI	0.00505725	0.00926328
11732126_x_at	UBB	Upregulated in MI	0.00014694	0.0016669
11729661_a_at	GLB1	Upregulated in MI	0.00069158	0.00284317
11717422_s_at	RBM8A	Upregulated in MI	0.0001261	0.00150508
11758158_s_at	FOXP1	Upregulated in MI	0.00078736	0.0030992
11743098_a_at	TARSL2	Upregulated in MI	0.00687991	0.01183985
11715501_s_at	IGFBP7	Upregulated in MI	9.2259E-05	0.00137774
11725969_a_at	THUMPD1	Upregulated in MI	0.0026334	0.00636836
11760913_at	ASAH2	Upregulated in MI	2.0029E-05	0.00061757
11718344_a_at	CNOT7	Upregulated in MI	0.00146865	0.0044541
11735389_at	CYLC2	Upregulated in MI	0.0036725	0.00780935
11747800_a_at	HIF1A	Upregulated in MI	0.04043046	0.0488865
11721215_a_at	TMEM106B	Upregulated in MI	0.00019823	0.0016669
11755052_s_at	GYPE	Upregulated in MI	0.00130072	0.00422165
11717433_a_at	ECHDC1	Upregulated in MI	4.8014E-05	0.00093501
11752628_a_at	SYNCRIP	Upregulated in MI	0.00376444	0.00791387
11747485_a_at	SR140	Upregulated in MI	0.00019823	0.0016669
11715657_a_at	GLB1	Upregulated in MI	0.00130072	0.00422165
11757402_s_at	MED13	Upregulated in MI	0.00026521	0.00192411
11730368_at	ZNF557	Upregulated in MI	0.02104398	0.028626
11718577_s_at	NET1	Upregulated in MI	0.00409148	0.00813896
11732339_at	BCL11A	Upregulated in MI	0.02493936	0.03283831
11744873_a_at	KLKP1	Upregulated in MI	0.00089487	0.00334446
11763952_at	–	Upregulated in MI	0.00030585	0.00205755
11726870_at	SETBP1	Upregulated in MI	0.0006064	0.00276999
11727433_s_at	NUTF2	Upregulated in MI	0.02423264	0.03248578
11726614_at	CDH2	Upregulated in MI	0.00505725	0.00926328
11744433_x_at	AMY2B	Upregulated in MI	3.4121E-05	0.00084164
11720250_a_at	RWDD1	Upregulated in MI	0.00010799	0.00137774
11724140_a_at	CRIPAK	Upregulated in MI	0.00053077	0.00252042
11754192_s_at	SFRS11	Upregulated in MI	2.3988E-05	0.00068274
11758666_s_at	LOC284861	Upregulated in MI	0.0006064	0.00276999
11764171_s_at	DCUN1D1	Upregulated in MI	0.00687991	0.01183985
11751513_a_at	TXNDC6	Upregulated in MI	0.0026334	0.00636836
11758715_s_at	DEFB126	Upregulated in MI	0.00502731	0.00926328
11755681_x_at	HMGB1	Upregulated in MI	9.4442E-06	0.00038514
11737234_s_at	LOC162632	Upregulated in MI	0.04365745	0.05177326
11751041_x_at	PCMTD2	Upregulated in MI	0.00146865	0.0044541
11720954_s_at	RPL30	Upregulated in MI	0.0026334	0.00636836
11732933_a_at	RUNX1	Upregulated in MI	0.00115015	0.00390417
11750545_a_at	CNOT7	Upregulated in MI	0.01229819	0.01880302
11716615_s_at	REEP5	Upregulated in MI	0.01477913	0.02163607
200037_PM_s_at	CBX3	Upregulated in MI	0.00561109	0.01012733
11749445_a_at	ARHGAP15	Upregulated in MI	0.00053077	0.00252042
11719713_a_at	PPM1B	Upregulated in MI	0.00455191	0.00871434
11725073_s_at	PHF17	Upregulated in MI	0.02104398	0.028626
11715490_s_at	AMY1A	Upregulated in MI	0.0026334	0.00636836
11757108_a_at	GSTTP1	Upregulated in MI	0.0036725	0.00780935
11758637_x_at	AMY1A	Upregulated in MI	0.0147944	0.02163607
11743386_s_at	PRPF40A	Upregulated in MI	0.00010799	0.00137774
11719932_x_at	KIAA0319L	Upregulated in MI	0.01229819	0.01880302
11750815_s_at	DDX5	Upregulated in MI	0.03190866	0.04002103
11761866_at	NCOA7	Upregulated in MI	0.01229819	0.01880302
11762842_s_at	PLEKHB2	Upregulated in MI	0.0036725	0.00780935
11758021_s_at	DDX3×	Upregulated in MI	0.00146865	0.0044541
11755779_a_at	ADAMTS20	Upregulated in MI	0.00294638	0.00689976
11734919_s_at	TCEA1	Upregulated in MI	0.00053077	0.00252042
11741476_x_at	MAP3K7	Upregulated in MI	0.00455191	0.00871434
11745795_s_at	DDX5	Upregulated in MI	0.01018421	0.01603471
11746794_a_at	C5orf33	Upregulated in MI	0.01617252	0.0232834
11758181_s_at	HMGB1	Upregulated in MI	0.01119828	0.01740908
11758811_x_at	HNRNPA1	Upregulated in MI	0.00329182	0.00729325
11761671_a_at	ETV7	Upregulated in MI	0.00186355	0.00526347
11758000_s_at	CXADR	Upregulated in MI	0.0036725	0.00780935
11733216_s_at	USP53	Upregulated in MI	0.01348981	0.02012593
200012_PM_x_at	RPL21	Upregulated in MI	5.1905E-06	0.0003841
11722616_at	UBLCP1	Upregulated in MI	0.00329182	0.00729325
11743186_a_at	KIAA1430	Upregulated in MI	0.01018421	0.01603471
11758697_x_at	RPL10A	Upregulated in MI	0.00294638	0.00689976
11758663_s_at	MATR3	Upregulated in MI	0.00062978	0.00284168
11739605_a_at	CCDC88A	Upregulated in MI	0.00839186	0.01392372
11718654_s_at	PKD2	Upregulated in MI	0.01119828	0.01740908
11740007_at	POLR3G	Upregulated in MI	0.00839186	0.01392372
11723448_x_at	MALL	Upregulated in MI	0.0026334	0.00636836
11725386_a_at	HOMER1	Upregulated in MI	0.00329182	0.00729325
11721237_a_at	LHFPL2	Upregulated in MI	0.00925052	0.01488126
11740255_x_at	UBE2NL	Upregulated in MI	0.00069158	0.00284317
11763843_a_at	UACA	Upregulated in MI	0.00069158	0.00284317
11755332_a_at	TJAP1	Upregulated in MI	0.00019528	0.0016669
11731400_s_at	TMCO1	Upregulated in MI	0.00089487	0.00334446
11755203_x_at	RPL21	Upregulated in MI	7.8646E-05	0.00137774
11748647_a_at	PTPRR	Upregulated in MI	0.02710422	0.03543661
11751975_a_at	SGIP1	Upregulated in MI	0.0036725	0.00780935
11739563_a_at	ITPR1	Upregulated in MI	0.00010799	0.00137774
11719614_a_at	LARP4	Upregulated in MI	0.00030585	0.00205755
11722359_x_at	EPB41L2	Upregulated in MI	0.00146865	0.0044541
11754410_s_at	APLNR	Upregulated in MI	0.00235021	0.00608096
11739606_x_at	CCDC88A	Upregulated in MI	0.00505725	0.00926328
11737468_a_at	PDC	Upregulated in MI	0.00409148	0.00813896
11732569_at	SLCO1B3	Upregulated in MI	0.04365745	0.05177326
11733180_a_at	ETV1	Upregulated in MI	0.02493936	0.03283831
11742053_a_at	COG5	Upregulated in MI	0.05074665	0.05885975
11732720_a_at	EREG	Upregulated in MI	0.00376444	0.00791387
11722645_s_at	ZNHIT6	Upregulated in MI	0.02104398	0.028626
11722842_s_at	ENAH	Upregulated in MI	1.145E-05	0.00038514
11746051_a_at	HP1BP3	Upregulated in MI	0.00666163	0.01173715
11758520_s_at	GUCY1A3	Upregulated in MI	0.00209437	0.00574011
11751517_a_at	PRKAA1	Upregulated in MI	0.00235021	0.00608096
11757817_s_at	BASP1	Upregulated in MI	0.00165565	0.00486183
11719053_s_at	CEP350	Upregulated in MI	0.0062173	0.0110067
11763534_x_at	CNTNAP3	Upregulated in MI	0.00235021	0.00608096
11715329_at	SLC6A15	Upregulated in MI	0.00069158	0.00284317
11761881_at	ZNF33A	Upregulated in MI	0.00146865	0.0044541
11723314_x_at	PXMP2	Upregulated in MI	2.476E-07	9.1611E-05
11758802_a_at	ENY2	Upregulated in MI	0.00069158	0.00284317
11758108_s_at	EFEMP1	Upregulated in MI	0.0027968	0.00667624
11739011_s_at	PAFAH1B1	Upregulated in MI	0.0026334	0.00636836
11749062_a_at	ERG	Upregulated in MI	0.00103627	0.00368673
11761958_s_at	TRA@	Upregulated in MI	0.01348981	0.02012593
11753646_x_at	CFL1	Upregulated in MI	0.00455191	0.00871434
11723447_at	MALL	Upregulated in MI	0.00053077	0.00252042
11725729_s_at	C1orf56	Upregulated in MI	0.00040442	0.00241348
11725658_a_at	MTFR1	Upregulated in MI	0.00925052	0.01488126
11725496_a_at	AGPAT9	Upregulated in MI	0.00687991	0.01183985
11735535_at	ZNF660	Upregulated in MI	0.00186355	0.00526347
11754898_a_at	ZNF573	Upregulated in MI	0.00839186	0.01392372
11719509_a_at	CSRP2BP	Upregulated in MI	0.00039848	0.00241348
11729721_s_at	LILRB3	Upregulated in MI	5.1905E-06	0.0003841
11728429_a_at	LCOR	Upregulated in MI	4.053E-05	0.00088212
11750795_a_at	KLHL1	Upregulated in MI	1.145E-05	0.00038514
11754487_x_at	C5orf33	Upregulated in MI	0.01018421	0.01603471
11727856_s_at	NUP50	Upregulated in MI	0.00069158	0.00284317
11732303_a_at	CREB1	Upregulated in MI	0.00035204	0.00224576
11738720_s_at	OR2T3	Upregulated in MI	0.00209437	0.00574011
11718993_at	CRKL	Upregulated in MI	0.00409148	0.00813896
11727390_a_at	STEAP2	Upregulated in MI	0.00121855	0.00406184
11728769_at	ST6GALNAC3	Upregulated in MI	0.00760327	0.0128457
11742385_s_at	OR8B2	Upregulated in MI	0.00240361	0.00617593
11756285_s_at	IGF2BP3	Upregulated in MI	0.02292193	0.03084041
11722814_s_at	RANBP2	Upregulated in MI	0.00046374	0.00245118
11751269_a_at	SUPT3H	Upregulated in MI	0.00455191	0.00871434
11763119_x_at	–	Upregulated in MI	0.01617252	0.0232834
11721835_s_at	TMEM14B	Upregulated in MI	0.00186355	0.00526347
11736501_x_at	SS18	Upregulated in MI	0.02292193	0.03084041
11717574_s_at	PFN1	Upregulated in MI	0.00165565	0.00486183
11757274_s_at	ARGLU1	Upregulated in MI	0.00030585	0.00205755
11729916_s_at	ARL5B	Upregulated in MI	0.01929797	0.0270464
11721216_s_at	TMEM106B	Upregulated in MI	0.00017084	0.0016669
11759049_at	ACSS3	Upregulated in MI	0.08324897	0.09305777
11736190_a_at	OGN	Upregulated in MI	0.01929797	0.0270464
11734314_at	SPTA1	Upregulated in MI	0.00089487	0.00334446
AFFX-HUMGAPDH/M33197_	GAPDH	Upregulated in MI	0.02942452	0.03767153
11753680_x_at	RPL21	Upregulated in MI	4.053E-05	0.00088212
11738693_at	OR13C3	Upregulated in MI	0.01348981	0.02012593
11746970_at	NPY6R	Upregulated in MI	0.04365745	0.05177326
11722149_a_at	YTHDC2	Upregulated in MI	0.05462796	0.06316358
11748766_a_at	FBXW7	Upregulated in MI	0.00022952	0.00176918
11744244_a_at	MASP1	Upregulated in MI	0.00235021	0.00608096
11759047_x_at	ABCB1	Upregulated in MI	0.00146865	0.0044541
11733995_x_at	C5orf33	Upregulated in MI	0.00046374	0.00245118
11719660_at	ATP1A2	Upregulated in MI	0.00069158	0.00284317
11732982_at	OR2J2	Upregulated in MI	0.0026334	0.00636836
11720945_x_at	SNRPA1	Upregulated in MI	0.05534295	0.06379094
11759697_at	SLITRK3	Upregulated in MI	0.00209437	0.00574011
11757257_at	PISRT1	Upregulated in MI	0.04365745	0.05177326
11728052_s_at	FAM126B	Upregulated in MI	0.01929797	0.0270464
11728076_at	HDAC9	Upregulated in MI	0.01980258	0.02764888
11742048_at	ITGB1	Upregulated in MI	0.00329182	0.00729325
11758636_s_at	ASPN	Upregulated in MI	0.00409148	0.00813896
11756080_s_at	NUS1	Upregulated in MI	0.05074665	0.05885975
11730843_a_at	MXI1	Upregulated in MI	0.00265809	0.00638632
11739731_s_at	CSNK1G1	Upregulated in MI	0.00505725	0.00926328
11719476_at	C20orf108	Upregulated in MI	0.02837207	0.03696361
11722845_s_at	UBE2R2	Upregulated in MI	0.03066104	0.03911925
11752838_s_at	CIDEB	Upregulated in MI	0.05074665	0.05885975
11753282_a_at	CMTM4	Upregulated in MI	0.02493936	0.03283831
11759361_at	SHOX	Upregulated in MI	0.00030585	0.00205755
11742378_a_at	AKR1B10	Upregulated in MI	0.01348981	0.02012593
11757489_x_at	RPL22	Upregulated in MI	0.00078736	0.0030992
11720443_s_at	BAZ1A	Upregulated in MI	0.03740276	0.04597682
11756351_x_at	SOD1	Upregulated in MI	0.00069158	0.00284317
11716368_x_at	PRR13	Upregulated in MI	0.00019823	0.0016669
11741875_x_at	AKTIP	Upregulated in MI	0.02104398	0.028626
11749630_a_at	KRR1	Upregulated in MI	0.02493936	0.03283831
11758560_s_at	HERC4	Upregulated in MI	0.00101536	0.00364739
11738204_a_at	SPAM1	Upregulated in MI	0.07779419	0.08775564
11757384_x_at	UROD	Upregulated in MI	0.00022952	0.00176918
11721520_at	ZDHHC17	Upregulated in MI	0.04709299	0.05549174
11753061_a_at	SLFN5	Upregulated in MI	0.0026334	0.00636836
11728110_at	GRIP1	Upregulated in MI	0.00505725	0.00926328
11722667_a_at	MAPT	Upregulated in MI	0.00687991	0.01183985
11718781_s_at	SSBP2	Upregulated in MI	2.865E-05	0.00075718
11750759_at	CES4	Upregulated in MI	0.00760327	0.0128457
11737583_s_at	SGCD	Upregulated in MI	0.00046374	0.00245118
11727015_s_at	PAPPA	Upregulated in MI	0.00186355	0.00526347
11728682_at	KRR1	Upregulated in MI	0.00329182	0.00729325
11737293_at	TACR3	Upregulated in MI	0.02104398	0.028626
11746047_x_at	KGFLP2	Upregulated in MI	0.00409148	0.00813896
11734530_x_at	HLA-F	Upregulated in MI	9.4442E-06	0.00038514
11723507_s_at	ZNF609	Upregulated in MI	0.05074665	0.05885975
11742902_s_at	AP3S1	Upregulated in MI	0.08324897	0.09305777
11741095_at	CORO2A	Upregulated in MI	0.00839186	0.01392372
11724290_x_at	ZNF641	Upregulated in MI	0.01119828	0.01740908
11738606_a_at	KCTD16	Upregulated in MI	0.00115015	0.00390417
11735483_at	LANCL3	Upregulated in MI	0.00925052	0.01488126
11739334_a_at	PTPRC	Upregulated in MI	0.0062173	0.0110067
11735206_at	MMAA	Upregulated in MI	0.00053077	0.00252042
11741856_s_at	LOC653501	Upregulated in MI	0.00235021	0.00608096
11735379_a_at	KIAA1009	Upregulated in MI	0.00329182	0.00729325
11719268_at	TNNC1	Upregulated in MI	0.03456513	0.04306093
11763439_s_at	HNRNPU	Upregulated in MI	0.00040442	0.00241348
11732224_a_at	ZNF664	Upregulated in MI	0.02942452	0.03767153
11730746_s_at	PAIP2	Upregulated in MI	0.00035204	0.00224576
11744413_x_at	HSPA1A	Upregulated in MI	0.01348981	0.02012593
11744535_s_at	TCEB1	Upregulated in MI	0.00925052	0.01488126
11728719_a_at	LTBP1	Upregulated in MI	0.0357177	0.04434747
11741101_a_at	ZNF655	Upregulated in MI	0.00505725	0.00926328
11723042_at	UBE2D1	Upregulated in MI	0.00455191	0.00871434
11716750_a_at	CD99L2	Upregulated in MI	0.01348981	0.02012593
11754732_a_at	CNDP1	Upregulated in MI	0.00019823	0.0016669
11740403_at	C12orf69	Upregulated in MI	0.0176765	0.02515502
11761250_x_at	ARL5A	Upregulated in MI	0.00455191	0.00871434
11763585_s_at	TMPO	Upregulated in MI	0.08900096	0.09859388
11738200_a_at	CCDC102B	Upregulated in MI	0.02942452	0.03767153
11718702_a_at	ARFIP1	Upregulated in MI	0.0631128	0.0718515
11747428_a_at	CDK20	Upregulated in MI	0.00329182	0.00729325
11745483_s_at	BECN1	Upregulated in MI	0.00026521	0.00192411
11730250_a_at	LNX1	Upregulated in MI	0.03190866	0.04002103
11716587_at	AXL	Upregulated in MI	0.00409148	0.00813896
11758860_at	HNRNPU	Upregulated in MI	0.01229819	0.01880302
11734908_a_at	CADPS2	Upregulated in MI	0.00329182	0.00729325
11734244_a_at	ATG10	Upregulated in MI	0.01617252	0.0232834
11728312_at	ZNF148	Upregulated in MI	0.03190866	0.04002103
11739159_at	FAM8A1	Upregulated in MI	0.00146865	0.0044541
11740664_a_at	GPRC6A	Upregulated in MI	0.00209437	0.00574011
11757874_x_at	PFDN1	Upregulated in MI	0.00760327	0.0128457
11756150_at	B2M	Upregulated in MI	0.02493936	0.03283831
11757936_s_at	GCSH	Upregulated in MI	0.00046374	0.00245118
11715593_s_at	YWHAH	Upregulated in MI	0.04043046	0.0488865
11729688_s_at	LYRM7	Upregulated in MI	0.00046374	0.00245118
11742446_s_at	FOXD4L2	Upregulated in MI	0.00186355	0.00526347
11759938_a_at	ITFG2	Upregulated in MI	0.0062173	0.0110067
11754680_a_at	MAP3K2	Upregulated in MI	0.00235021	0.00608096
11717578_a_at	VPS26A	Upregulated in MI	0.03190866	0.04002103
11744671_x_at	CTBP2	Upregulated in MI	0.0631128	0.0718515
11751946_a_at	ARHGAP21	Upregulated in MI	0.00130072	0.00422165
11729687_at	LYRM7	Upregulated in MI	0.02104398	0.028626
11750160_a_at	LSM14A	Upregulated in MI	0.02942452	0.03767153
11727125_a_at	PVRL3	Upregulated in MI	0.03456513	0.04306093
11722445_a_at	TRAPPC5	Upregulated in MI	1.145E-05	0.00038514
11757916_s_at	TBX3	Upregulated in MI	0.0036725	0.00780935
11739487_s_at	SUZ12	Upregulated in MI	0.00395253	0.00813896
11756151_x_at	B2M	Upregulated in MI	0.01477913	0.02163607
11743427_at	AHCYL1	Upregulated in MI	0.03190866	0.04002103
11733078_a_at	PPP1R12A	Upregulated in MI	0.00925052	0.01488126
11760812_at	C10orf46	Upregulated in MI	0.04709299	0.05549174
11758911_at	DYRK2	Upregulated in MI	0.08900096	0.09859388
11724018_a_at	LDHC	Upregulated in MI	0.00019823	0.0016669
11753232_a_at	NDRG1	Upregulated in MI	0.00115015	0.00390417
11729110_s_at	ADAMDEC1	Upregulated in MI	0.05074665	0.05885975
11757659_x_at	RPL12	Upregulated in MI	0.00101536	0.00364739
11754221_s_at	FAM104B	Upregulated in MI	0.00235021	0.00608096
11753454_a_at	PRKG1	Upregulated in MI	0.03740276	0.04597682
11715662_x_at	TMEM189	Upregulated in MI	0.00409148	0.00813896
11760194_at	LRRIQ3	Upregulated in MI	0.00329182	0.00729325
11761070_at	MRPL20	Upregulated in MI	0.01018421	0.01603471
11750330_a_at	MYOCD	Upregulated in MI	0.01477913	0.02163607
11728272_x_at	ZNF330	Upregulated in MI	0.00561109	0.01012733
11743661_a_at	MBNL1	Upregulated in MI	0.00414689	0.00820508
11731899_s_at	PPAT	Upregulated in MI	0.08883741	0.09859388
11725485_at	DIRC2	Upregulated in MI	0.0176765	0.02515502
11758273_s_at	ARF6	Upregulated in MI	0.00046374	0.00245118
11751364_a_at	SLC47A1	Upregulated in MI	0.0026334	0.00636836
11740734_a_at	A2BP1	Upregulated in MI	0.02942452	0.03767153
11743792_a_at	TTC39C	Upregulated in MI	0.02710422	0.03543661
AFFX-r2-Ec-bioB-5_at	–	Upregulated in MI	0.00019823	0.0016669
11752951_x_at	RPL15	Upregulated in MI	0.04365745	0.05177326
11720802_s_at	BIN3	Downregulatedin MI	9.2259E-05	0.00137774
11760353_at	SAFB2	Downregulatedin MI	0.00687991	0.01183985
11754373_a_at	MARK3	Downregulatedin MI	0.00046374	0.00245118
11730830_x_at	AMPD2	Downregulatedin MI	0.00505725	0.00926328
11737523_x_at	VSIG8	Downregulatedin MI	1.145E-05	0.00038514
11759492_at	RPS27L	Downregulatedin MI	0.00235021	0.00608096
11728009_at	VPREB3	Downregulatedin MI	0.00146865	0.0044541
11730408_a_at	C19orf33	Downregulatedin MI	0.0385022	0.04717157
11728044_a_at	C14orf126	Downregulatedin MI	0.00875884	0.01446773
11763618_a_at	SNAPC4	Downregulatedin MI	0.00026521	0.00192411
11716415_s_at	NDUFB8	Downregulatedin MI	0.00165565	0.00486183
11737238_s_at	EOMES	Downregulatedin MI	0.0062173	0.0110067
11760953_x_at	UBXN11	Downregulatedin MI	0.00022952	0.00176918
11746163_a_at	LARP4B	Downregulatedin MI	0.04043046	0.0488865
11724561_x_at	TSR2	Downregulatedin MI	0.02292193	0.03084041
11735855_at	TNP2	Downregulatedin MI	0.00409148	0.00813896
11757649_a_at	TIMM16	Downregulatedin MI	7.8646E-05	0.00137774
11732524_a_at	CHKB-CPT1B	Downregulatedin MI	0.00358451	0.00780935
11736945_a_at	HIPK4	Downregulatedin MI	0.00040442	0.00241348
11716547_s_at	TLR9	Downregulatedin MI	0.01477913	0.02163607
11746635_a_at	LEF1	Downregulatedin MI	0.00089487	0.00334446
11744612_a_at	NUDCD1	Downregulatedin MI	0.00121855	0.00406184
11731356_a_at	IP6K3	Downregulatedin MI	0.00078736	0.0030992
11728896_a_at	LRP8	Downregulatedin MI	0.01018421	0.01603471
11759458_at	LOC285501	Downregulatedin MI	0.01617252	0.0232834
11762010_a_at	GRAMD1B	Downregulatedin MI	0.00019823	0.0016669
11757381_x_at	GTF2A2	Downregulatedin MI	0.0728868	0.0824713
11743193_a_at	PARD6G	Downregulatedin MI	0.00019823	0.0016669
11726367_a_at	ERICH1	Downregulatedin MI	0.03740276	0.04597682
11750342_a_at	FRMPD1	Downregulatedin MI	0.08324897	0.09305777
11744231_a_at	MAPK7	Downregulatedin MI	0.00294638	0.00689976
11724255_a_at	OAS1	Downregulatedin MI	0.0176765	0.02515502
11737305_a_at	FAM166A	Downregulatedin MI	0.00561109	0.01012733
11733958_a_at	GTPBP3	Downregulatedin MI	0.00089487	0.00334446
11739429_a_at	ZDHHC24	Downregulatedin MI	0.01900683	0.02694455
11751172_a_at	TRIB3	Downregulatedin MI	0.05732237	0.06586732
11737677_at	BTBD18	Downregulatedin MI	0.02104398	0.028626
11725393_s_at	MAK16	Downregulatedin MI	0.0631128	0.0718515
11744029_a_at	BBS4	Downregulatedin MI	0.04043046	0.0488865
11743134_x_at	FKBP8	Downregulatedin MI	0.00022952	0.00176918
11745187_a_at	BET1L	Downregulatedin MI	0.00925052	0.01488126
11737856_a_at	OPCML	Downregulatedin MI	0.00101536	0.00364739
11759126_a_at	THRA	Downregulatedin MI	0.04365745	0.05177326
11722129_at	FAM102B	Downregulatedin MI	0.00165565	0.00486183
11762149_at	C18orf45	Downregulatedin MI	0.00115015	0.00390417
11734407_a_at	MATN4	Downregulatedin MI	0.00760327	0.0128457
11730872_x_at	RASSF5	Downregulatedin MI	0.00409148	0.00813896
11753413_x_at	DLK1	Downregulatedin MI	0.07262653	0.08242889

Gene Ontology (GO) analysis of the DEGs was performed using DAVID Bioinformatics tools [3] (http://david.abcc.ncifcrf.gov/). The GO results for the down-regulated transcripts were not enriched for any GO terms. The GO analysis revealed biological processes (Table 6).

**Table 6:** Gene ontology of upregulated genes.

**Category**	**Term**	**Count**	**%**	***p*****-value**	**Genes**	**List Total**	**Pop Hits**	**Pop Total**	**Fold Enrichmen**	**tBonferroni**	**Benjamini**	**FDR**
GOTERM_BP_FAT	GO:0070647~protein modification by small protein conjugation or removal	12	4.109589	4.96E-05	ENY2, UBE2N, SUZ12, SUPT3H, ATG10, FBXW7, UBE3A, UBB, UBE2D1, TMEM189, FBXW11, LNX1	216	160	13528	4.697222222	0.08427547	0.084275	0.084054
GOTERM_BP_FAT	GO:0032446~protein modification by small protein conjugation	10	3.4246575	2.56E-04	UBE2N, SUZ12, ATG10, FBXW7, UBE3A, UBB, UBE2D1, TMEM189, FBXW11, LNX1	216	132	13528	4.744668911	0.365690828	0.203565	0.433849
GOTERM_BP_FAT	GO:0016567~protein ubiquitination	9	3.0821918	6.19E-04	UBE2N, SUZ12, FBXW7, UBE3A, UBB, UBE2D1, TMEM189, FBXW11, LNX1	216	119	13528	4.736694678	0.666812995	0.30674	1.044247
GOTERM_BP_FAT	GO:0006940~regulation of smooth muscle contraction	5	1.7123288	0.00299912	TACR3, MYOCD, GUCY1A3, ATP1A2, SOD1	216	38	13528	8.240740741	0.995162875	0.736278	4.964734
GOTERM_BP_FAT	GO:0051147~regulation of muscle cell differentiation	5	1.7123288	0.00330086	TBX3, MYOCD, EREG, UBB, HDAC9	216	39	13528	8.029439696	0.99717351	0.690793	5.451184
GOTERM_BP_FAT	GO:0006414~translational elongation	7	2.3972603	0.00544585	RPL30, RPL22, RPL21, RPL15, UBB, RPL10A, RPL12	216	101	13528	4.3406674	0.999938274	0.801202	8.842269
GOTERM_BP_FAT	GO:0006937~regulation of muscle contraction	6	2.0547945	0.00571597	TACR3, MYOCD, TNNC1, GUCY1A3, ATP1A2, SOD1	216	72	13528	5.219135802	0.999961887	0.766265	9.261108
GOTERM_BP_FAT	GO:0048742~regulation of skeletal muscle fiber development	4	1.369863	0.00703496	TBX3, MYOCD, UBB, HDAC9	216	25	13528	10.02074074	0.999996388	0.791204	11.28038
GOTERM_BP_FAT	GO:0007507~heart development	10	3.4246575	0.00750053	CRKL, TBX3, MYOCD, HEXIM1, TNNC1, PKD2, HDAC9, CXADR, ITGB1, FOXP1	216	215	13528	2.913006029	0.999998429	0.773463	11.98298
GOTERM_BP_FAT	GO:0016202~regulation of striated muscle tissue development	5	1.7123288	0.00807194	TBX3, MYOCD, UBB, HDAC9, CXADR	216	50	13528	6.262962963	0.999999435	0.762736	12.83814
GOTERM_BP_FAT	GO:0048634~regulation of muscle development	5	1.7123288	0.00865211	TBX3, MYOCD, UBB, HDAC9, CXADR	216	51	13528	6.140159768	0.9999998	0.753948	13.69842
GOTERM_BP_FAT	GO:0048534~hemopoietic or lymphoid organ development	11	3.7671233	0.00874561	PTPRC, CRKL, RPL22, BCL11A, TCEA1, SPTA1, HDAC9, SOD1, RUNX1, ITGB1, FOXP1	216	260	13528	2.6497151	0.999999831	0.727281	13.83632
GOTERM_BP_FAT	GO:0030036~actin cytoskeleton organization	10	3.4246575	0.01017938	EPB41L2, PFN1, CALD1, CFL1, PAFAH1B1, ARF6, SPTA1, PRKG1, ITGB1, KLHL1	216	226	13528	2.77122255	0.999999987	0.752662	15.92501
GOTERM_BP_FAT	GO:0048641~regulation of skeletal muscle tissue development	4	1.369863	0.01066911	TBX3, MYOCD, UBB, HDAC9	216	29	13528	8.638569604	0.999999995	0.743329	16.62747
GOTERM_BP_FAT	GO:0045935~positive regulation of nucleobase, nucleoside, nucleotide and nuc	19	6.5068493	0.01072364	ENY2, BMP3, PTPRC, TBX3, GRIP1, CREB1, MED13, DDX5, CNOT7, UBE2N, YWHAH, HIF1A, MYOCD, EREG, ZNF148, GU	216	624	13528	1.906991928	0.999999995	0.720797	16.70534
GOTERM_CC_FAT	GO:0030529~ribonucleoprotein complex	19	6.7857143	9.63E-04	KRR1, SNRPA1, ERG, RPL15, SYNCRIP, HSPA1A, DDX5, HNRNPA1, HNRNPU, MRPL20, RPL30, RBM8A, RPL22, PNRC2, R	197	515	12782	2.393750924	0.246766301	0.246766	1.275853
GOTERM_CC_FAT	GO:0005829~cytosol	36	12.857143	9.94E-04	NAMPT, ENAH, UBE3A, GRIP1, RPL15, MAP3K7, RPL30, MAP3K2, MAPT, GSTZ1, GUCY1A3, PAFAH1B1, PPP3CA, RPL12	197	1330	12782	1.756238312	0.253459145	0.135974	1.315771
GOTERM_CC_FAT	GO:0031981~nuclear lumen	37	13.214286	0.00232476	ENY2, SUPT3H, HMGB1, SYNCRIP, ZNF655, CNOT7, ZNF330, CORO2A, DDX3×, RBM8A, ZNF148, NUP50, TCEA1, UBE2D	197	1450	12782	1.655641519	0.495544858	0.203949	3.053045
GOTERM_CC_FAT	GO:0005794~Golgi apparatus	25	8.9285714	0.00378136	CCDC88A, AIMP1, BECN1, PTPRR, AP3S1, ARF6, ARFIP1, CXADR, PRKG1, GCC2, TJAP1, B2M, ARHGAP21, PNPLA8, ZDHH	197	872	12782	1.860184883	0.671699641	0.243049	4.921769
GOTERM_CC_FAT	GO:0005654~nucleoplasm	25	8.9285714	0.00436162	ENY2, HMGB1, SUPT3H, SYNCRIP, CNOT7, CORO2A, DDX3×, RBM8A, NUP50, TCEA1, UBE2D1, KPNB1, PRPF40A, POLR	197	882	12782	1.839094352	0.72338246	0.22665	5.656882
GOTERM_CC_FAT	GO:0030864~cortical actin cytoskeleton	4	1.4285714	0.0087726	EPB41L2, CALD1, CFL1, SPTA1	197	28	12782	9.269035533	0.925019331	0.350631	11.07554
GOTERM_CC_FAT	GO:0005635~nuclear envelope	9	3.2142857	0.01377595	UACA, NUP50, CBX3, PAFAH1B1, TMPO, RANBP2, KPNB1, MATR3, ITPR1	197	205	12782	2.848532871	0.983063526	0.441562	16.87269
GOTERM_CC_FAT	GO:0070013~intracellular organelle lumen	39	13.928571	0.02000434	ENY2, SUPT3H, HMGB1, SYNCRIP, ZNF655, CNOT7, ZNF330, MRPL20, CORO2A, DDX3×, RBM8A, ZNF148, NUP50, TCEA	197	1779	12782	1.42239837	0.997370329	0.524131	23.60067
GOTERM_CC_FAT	GO:0044451~nucleoplasm part	16	5.7142857	0.02385422	ENY2, POLR3G, SUPT3H, CTBP2, CREB1, YY1, MED13, CNOT7, SUZ12, CORO2A, PHF17, HIF1A, DDX3×, RBM8A, HDAC9	197	555	12782	1.870508072	0.99917336	0.545557	27.50364
GOTERM_CC_FAT	GO:0031965~nuclear membrane	5	1.7857143	0.02568978	CBX3, PAFAH1B1, TMPO, MATR3, ITPR1	197	73	12782	4.444058132	0.999524672	0.534736	29.29883
GOTERM_CC_FAT	GO:0043233~organelle lumen	39	13.928571	0.0276102	ENY2, SUPT3H, HMGB1, SYNCRIP, ZNF655, CNOT7, ZNF330, MRPL20, CORO2A, DDX3×, RBM8A, ZNF148, NUP50, TCEA	197	1820	12782	1.39035533	0.999733881	0.526841	31.13293
GOTERM_CC_FAT	GO:0030054~cell junction	15	5.3571429	0.02854188	ARHGAP21, PTPRC, ENAH, CTBP2, CADPS2, GRIP1, PVRL3, CD99L2, ABCB1, CDH2, HOMER1, CXADR, ITGB1, RIMS1, TJA	197	518	12782	1.878858554	0.999799239	0.508085	32.00679
GOTERM_CC_FAT	GO:0044445~cytosolic part	7	2.5	0.02964735	RPL30, PFDN1, UACA, RPL22, RPL21, GUCY1A3, UBB	197	152	12782	2.988044349	0.999856352	0.4937	33.03034
GOTERM_CC_FAT	GO:0005912~adherens junction	7	2.5	0.03218777	PTPRC, ENAH, PVRL3, ABCB1, CDH2, CXADR, ITGB1	197	155	12782	2.930211233	0.999933536	0.496948	35.32875
GOTERM_CC_FAT	GO:0031974~membrane-enclosed lumen	39	13.928571	0.03603007	ENY2, SUPT3H, HMGB1, SYNCRIP, ZNF655, CNOT7, ZNF330, MRPL20, CORO2A, DDX3×, RBM8A, ZNF148, NUP50, TCEA	197	1856	12782	1.363387231	0.999979362	0.512871	38.66672
GOTERM_MF_FAT	GO:0003723~RNA binding	23	8.2142857	0.0016071	KRR1, SNRPA1, AIMP1, CPEB2, RPL15, SYNCRIP, MBNL1, IGF2BP3, DDX5, HNRNPA1, HNRNPU, MRPL20, RPL30, LARP4	201	718	12983	2.069104339	0.477004104	0.477004	2.220802
GOTERM_MF_FAT	GO:0016881~acid-amino acid ligase activity	10	3.5714286	0.00396374	UBE2N, AKTIP, UBE3A, HERC4, UBE2NL, UBE2D1, TMEM189, FBXW11, LNX1, UBE2R2	201	201	12983	3.213534318	0.798217111	0.550797	5.394694
GOTERM_MF_FAT	GO:0019787~small conjugating protein ligase activity	9	3.2142857	0.00418513	UBE2N, AKTIP, UBE3A, UBE2NL, UBE2D1, TMEM189, FBXW11, LNX1, UBE2R2	201	166	12983	3.501978061	0.815507665	0.43072	5.687888
GOTERM_MF_FAT	GO:0003702~RNA polymerase II transcription factor activity	11	3.9285714	0.00457311	SUPT3H, ETV7, HIF1A, TBX3, ZNF148, CREB1, TCEA1, MED13, TCEB1, LCOR, FOXP1	201	244	12983	2.911936221	0.842320571	0.36985	6.199674
GOTERM_MF_FAT	GO:0016879~ligase activity, forming carbon-nitrogen bonds	10	3.5714286	0.00955683	UBE2N, AKTIP, UBE3A, HERC4, UBE2NL, UBE2D1, TMEM189, FBXW11, LNX1, UBE2R2	201	231	12983	2.796192199	0.979140063	0.538828	12.54851
GOTERM_MF_FAT	GO:0003735~structural constituent of ribosome	8	2.8571429	0.01527537	RPL30, RPL22, RPL21, RPL15, UBB, RPL10A, RPL12, MRPL20	201	168	12983	3.075811419	0.997977614	0.644387	19.34096
GOTERM_MF_FAT	GO:0016566~specific transcriptional repressor activity	4	1.4285714	0.01770409	HEXIM1, YY1, HDAC9, FOXP1	201	36	12983	7.176893311	0.999252415	0.642414	22.07479
GOTERM_MF_FAT	GO:0030528~transcription regulator activity	34	12.142857	0.02428221	ENY2, SUPT3H, ETV7, GRIP1, CBX3, CNOT7, MXI1, MYOCD, HEXIM1, ZNF148, BCL11A, ETV1, TCEA1, ERG, ZNF33A, SSB	201	1512	12983	1.452466503	0.99995015	0.710127	29.05338
GOTERM_MF_FAT	GO:0003712~transcription cofactor activity	12	4.2857143	0.02515659	ENY2, SUPT3H, CTBP2, MYOCD, GRIP1, YY1, CREB1, BCL11A, MED13, HDAC9, DDX5, MXI1	201	363	12983	2.135274043	0.999965267	0.680458	29.936
GOTERM_MF_FAT	GO:0004842~ubiquitin-protein ligase activity	7	2.5	0.0262357	UBE2N, UBE3A, UBE2NL, UBE2D1, FBXW11, LNX1, UBE2R2	201	147	12983	3.075811419	0.999977772	0.657477	31.01122
GOTERM_MF_FAT	GO:0008134~transcription factor binding	15	5.3571429	0.02754274	ENY2, SUPT3H, HMGB1, CTBP2, GRIP1, YY1, CREB1, MED13, MXI1, DDX5, HIF1A, MYOCD, BCL11A, LCOR, HDAC9	201	513	12983	1.888656135	0.999987063	0.640565	32.29303
GOTERM_MF_FAT	GO:0003779~actin binding	11	3.9285714	0.02969715	EPB41L2, PFN1, CORO2A, ENAH, YWHAH, CCDC88A, TNNC1, CALD1, CFL1, SPTA1, KLHL1	201	326	12983	2.179486006	0.999994708	0.636668	34.3577
GOTERM_MF_FAT	GO:0019899~enzyme binding	15	5.3571429	0.03171181	PTPRC, CCDC88A, PTPRR, CBX3, CDH2, SOD1, RIMS1, PFN1, YWHAH, HIF1A, MAPT, PKD2, PAFAH1B1, RANBP2, HDAC9	201	523	12983	1.852544163	0.99999771	0.631749	36.23541
GOTERM_MF_FAT	GO:0008092~cytoskeletal protein binding	14	5	0.04821415	ENAH, CCDC88A, TNNC1, CALD1, KLHL1, EPB41L2, PFN1, CORO2A, YWHAH, MAPT, CFL1, PKD2, PAFAH1B1, SPTA1	201	504	12983	1.794223328	0.999999998	0.758878	49.84218
GOTERM_MF_FAT	GO:0016564~transcription repressor activity	10	3.5714286	0.05535048	CTBP2, TBX3, HEXIM1, ZNF148, YY1, BCL11A, CBX3, HDAC9, MXI1, FOXP1	201	316	12983	2.044051892	1	0.783426	54.84568

Clustering of genes were done by two methods, hierarchical and *k*-mean clustering. Hierarchical clustering with multiscale bootstrap resampling was done by Pvclust, an R statistical software package [4]. The Pvclust is an R package for assessing the uncertainty in hierarchical cluster analysis. For each cluster in hierarchical clustering, quantities called p-values are calculated via multiscale bootstrap resampling. The parameters (https://cran.r-project.org/web/packages/pvclust/pvclust.pdf) used here were 10000 bootstrap replications, cluster method: Ward algorithm and distance method: Euclidean. For the heat maps plot, we used log2 scale.

The *k*-mean clustering was performed by R (https://stat.ethz.ch/R-manual/R-devel/library/stats/html/kmeans.html), showing that the selection of features gave a higher accuracy than PAM alone. The genes were discriminated between the MI and non-MI vascular smooth muscle cells (VSMCs) samples (Table 7). A clustered result is shown in Fig. 2 of Ref. [1].

**Fig. 2:**
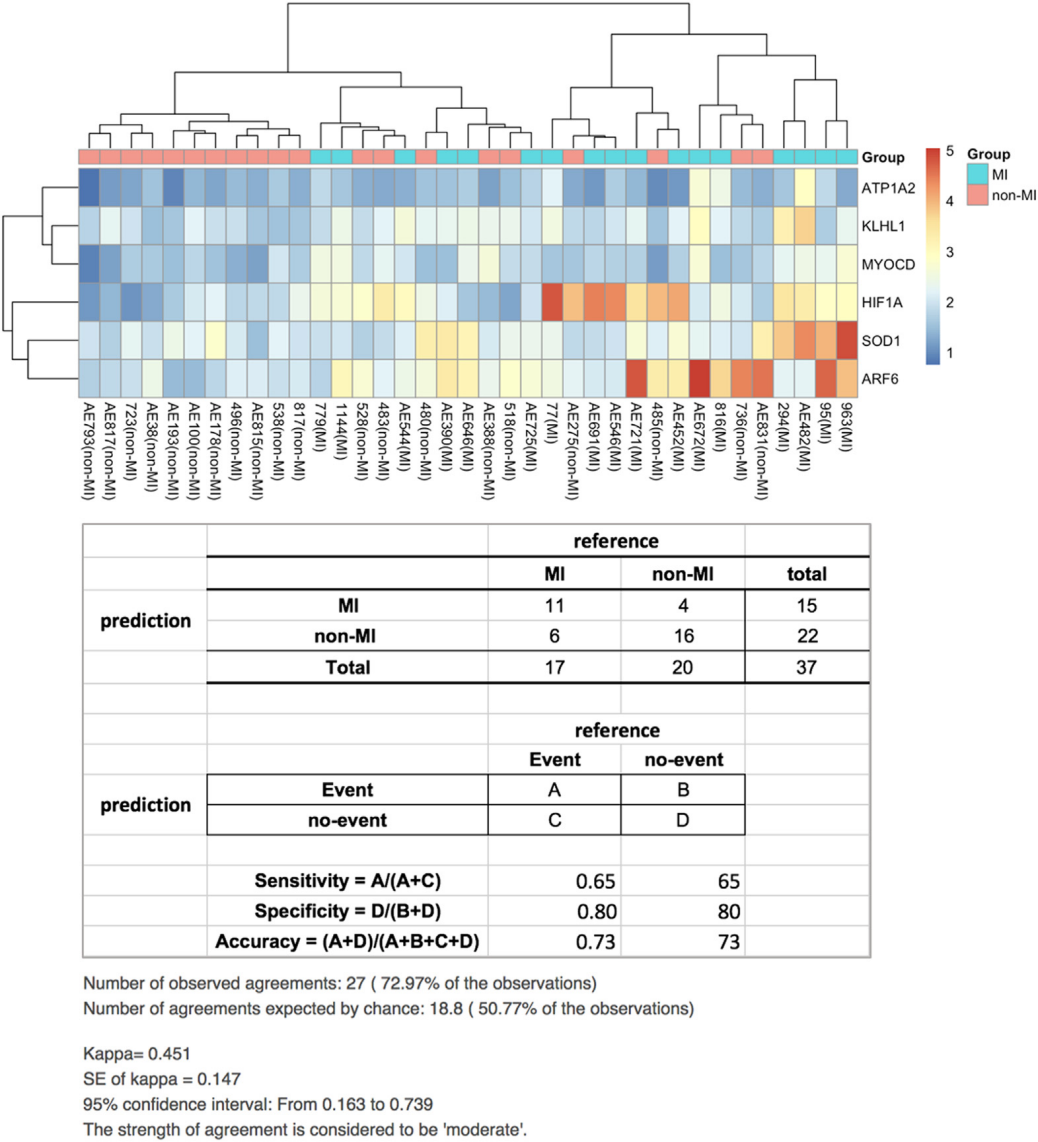
Hierarchical clustering on six RT-qPCR-based validation genes.

**Table 7:** Contingency table of prediction results from 21 genes.

		**Reference**		
		**MI**	**Non-MI**	**Total**
**Prediction**	**MI**	16	2	18
	**Non-MI**	1	18	19
	**Total**	17	20	37
			
		**Reference**		
		**Event**	**No-Event**	**Total**
**Prediction**	**Event**	A	B	A+B
	**No-Event**	C	D	C+D
	**Total**	A+C	B+D	A+B+C+D
	**Sensitivity = A/(A+C)**	0.941176	94	
	**Specificity = D/(B+D)**	0.9	90	
	**Accuracy = (A+D)/(A+B+C+D)**	0.918919	92	

### 1.3 Protein processing, electrostatic repulsion-hydrophilic interaction chromatography (ERLIC) and LC-MS/MS analysis using Q-Exactive mass spectrometer

Differential expressed proteins identified are shown in Table 8. Only peptides identified with strict spectral false discovery rate of less than 1% (q-value ≤ 0.01) were considered.

**Table 8:** Differentially expressed proteins.

### 1.4 Hierarchical cluster analysis of RT-qPCR-based detected genes

Using six RT-qPCR-supported genes as a representative gene classifier characterizing the differences between MI and non-MI aortic samples, hierarchical clustering was performed with multiscale bootstrap resampling by Pvclust. The result is shown in Fig. 2.

### 1.5 Transcriptomic and proteomic pathways analysis

Systemic evaluation was performed using IPA (www.ingenuity.com) to identify transcriptomic and proteomic pathways, and significantly enriched canonical pathways are shown in Table 9. An integrated transcriptome-proteome correlation is performed to identify common enriched pathway and molecule (Table 10).

**Table 9:** Pathway mapping of 370 transcripts (highlighted in light blue) and 94 proteins (highlighted in yellow).

**Table 10:** Pathway mapping of combined 21 gene signature and 94 proteins.

**Ingenuity Canonical Pathways**	**-log(p-value)**	**Ratio**	**Molecules**
Superoxide Radicals Degradation	3.1	0.4	CAT,SOD1
RhoGDI Signaling	2.11	0.046	ITGB1,RACK1,GDI2,ACTG1
Acetyl-CoA Biosynthesis III (from Citrate)	2.04	1	ACLY
Clathrin-mediated Endocytosis Signaling	1.9	0.04	ITGB1,UBB,ARF6,ACTG1
Amyotrophic Lateral Sclerosis Signaling	1.82	0.052	CAT,BID,SOD1
Paxillin Signaling	1.82	0.052	ITGB1,ARF6,ACTG1
Regulation of eIF4 and p70S6K Signaling	1.69	0.035	ITGB1,EIF3G,EIF3F,RPS4X
Actin Cytoskeleton Signaling	1.64	0.033	ITGB1,PFN1,SSH3,ACTG1
Mitochondrial Dysfunction	1.63	0.033	NDUFA9,NDUFV2,CAT,COX5A
Crosstalk between Dendritic Cells and Natural Killer Cells	1.61	0.074	CAMK2D,ACTG1
NRF2-mediated Oxidative Stress Response	1.57	0.032	UBB,CAT,SOD1,ACTG1

## 2 Experimental design, materials and methods

### 2.1 Sample collection

Aortic tissue samples were obtained from patients who presented with coronary artery disease undergoing coronary artery bypass graft (CABG) surgery at the National University Hospital of Singapore from 2009 to 2013. Patients underwent CABG either after a recent myocardial infarction (MI group) or as stable angina patients (non-MI group). An aortic punch tissue was collected at the time of proximal anastomosis between the aorta and saphenous vein grafts. The tissues from the aortic punch were immediately preserved on dry ice, and stored in liquid nitrogen tank. The study was approved by the National Healthcare Group Domain Specific Review Board (Tissue Bank registration: NUH/2009-0073), and written informed consent was obtained from all patients. The study protocol conforms to the ethical guidelines of the 1975 Declaration of Helsinki.

### 2.2 Sample grouping

**Fig. 3:**
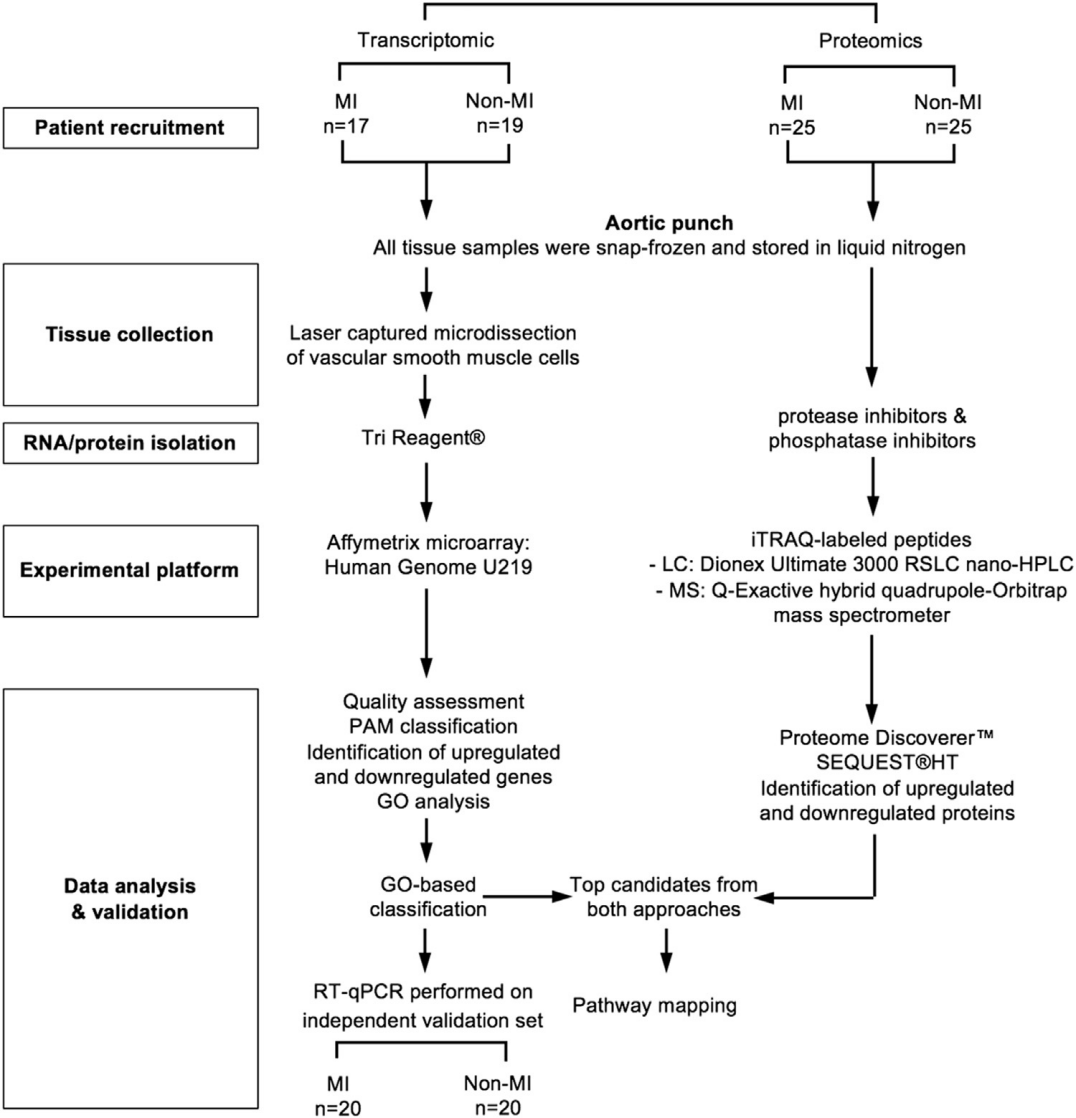
Work flow and study design.

17 MI and 19 non-MI samples were recruited for laser capture microdissection (LCM) and microarray profiling. The proteomic study included 25 MI and 25 non-MI samples. Four MI and six non-MI samples overlapped between the microarray and proteomic studies. RT-qPCR was done on an independent cohort of samples, including an additional 20 MI and 20 non-MI samples. A schematic of the design and workflow is presented in Fig. 3.

### 2.3 Sample processing

The protocols for (1) cryosectioning and staining of aortic tissue, (2) LCM of VSMCs, total RNA isolation and complementary DNA (cDNA) synthesis, and (3) protein processing, ERLIC and LC-MS/MS analysis using Q-Exactive mass spectrometer are described in our manuscript [1].

### 2.4 RT-qPCR on an independent cohort of MI and non-MI samples

The RT-qPCR protocol is described in our manuscript [1]. The primers for *ARF6, ATP1A2*, *GUCY1A3*, *HIF-1A*, *KLHL1*, *MYOCD*, *SOD1*, and *UBB* were obtained from the Primer Bank: *ARF6* forward primer 5′-GGGAAGGTGCTATCCAAAATCTT-3′ and reverse primer 5′-CACATCCCATACGTTGAACTTGA-3′; *ATP1A2* forward primer 5′-TCTATCCACGAGCGAGAAGAC-3′ and reverse primer 5′-CCATGTAGGCATTTTGAAAGGC-3′; *GUCY1A3* forward primer 5′-TCAGCCCTACTTGTTGTACTCC-3′ and reverse primer 5′-CAGAATAGCGATGTGGGAATCAC-3′; *HIF-1A* forward primer 5′-GAACGTCGAAAAGAAAAGTCTCG-3′ and reverse primer 5′-CCTTATCAAGATGCGAACTCACA-3′; *KLHL1* forward primer 5′-TCAGGCTCTGGGCGAAAAG-3′ and reverse primer 5′-AAAGTGCTCACACCGCTTCTC-3′; *MYOCD* forward primer 5′-ACGGATGCTTTTGCCTTTGAA-3′ and reverse primer 5′- AACCTGTCGAAGGGGTATCTG-3′; *SOD1* forward primer 5′-AAAGATGGTGTGGCCGATGT-3′ and reverse primer 5′-CAAGCCAAACGACTTCCAGC-3′; *UBB* forward primer 5′-GGTCCTGCGTCTGAGAGGT-3′ and reverse primer 5′-GGCCTTCACATTTTCGATGGT-3′.
